# *Clogmia albipunctata* (Nematocera; Psychodidae) as the Etiologic Agent of Myiasis: True or False?

**DOI:** 10.3390/diagnostics12092129

**Published:** 2022-09-01

**Authors:** Mohammad Akhoundi, Nambininiavo Marianne Ranorohasimanana, Sophie Brun, Catherine Kauffmann-Lacroix, Arezki Izri

**Affiliations:** 1 Parasitology-Mycology Department, Avicenne Hospital, AP-HP, Sorbonne Paris Nord University, 93009 Bobigny, France; 2 Laboratoire de Parasitologie et Mycologie Médicale, CHU de Poitiers, 86021 Poitiers, France; 3 Unité des Virus Émergents (UVE: Aix-Marseille Université-IRD 190-Inserm 1207-IHU Méditerranée Infection), 13008 Marseille, France

**Keywords:** *Clogmia albipunctata*, moth midge, non-hematophagous insect, myiasis, delusional parasitosis

## Abstract

*Clogmia albipunctata*, known as drain fly, is a non-hematophagous insect of the Psychodidae family with worldwide distribution, particularly in tropical and temperate areas. It can be found near sewer drains, sewage treatment plants, plant pots, swamps, and any other place containing decaying or moist organic matter. It has been introduced in several publications as the causative agent of myiasis in humans. A case presentation, together with a compilation of findings from a database, including 51 scientific publications in the literature, allowed us to overview critically in detail the variable aspects of epidemiology, life cycle, biology, and medical importance of this insect and its probable role in human myiasis. The absence of a precise definition of myiasis and the lack of incontestable epidemiological, entomological, and clinical evidence in the articles introducing *C. albipunctata* as a causative agent led us to interrogate its role in human myiasis. It is necessary to take into account this misinterpretation and make an accurate diagnosis based on the isolation of insect larvae from the corresponding lesion.

## 1. Introduction

*Clogmia* (*Telmatoscopus*) *albipunctata* Williston, 1893 are non-biting moth flies belonging to the Psychodidae family, which are known as drain or bathroom flies [1]. They are cosmopolitan species widespread across the world, particularly in tropical and subtropical countries [2,3,4]. They are not blood-sucking insects, but they may pose certain health risks to humans, as they commonly occur in large numbers in synanthropic habitats and are mainly considered nuisance pests.

### 1.1. Life Cycle and Ecology

*Clogmia albipunctata* (Nematocera; Psychodidae) are small (3–5 mm) holometabolous insects possessing a four-stage life cycle including egg, larva (four instars), pupa, and adult with a growth period of 27 ± 5 days from egg to adult [5]. Rising temperature and humidity affect the life cycle period and accelerate their growth. A female can lay from 200 to 300 eggs in her lifetime in sludge around sewage and drainage areas [2,6]. Eggs are small and transparent, no larger than 1 mm and hatch within 32–48 h [7]. Larvae are copro-saprophagous with chewing mouthparts living in aquatic environments, feeding mainly on organic decaying matters [8]. They have a respiratory siphon with or without fixing hooks to breathe in the aquatic areas. They turn into pupa and then adult after 18 and 5 days, respectively [5]. The adults commonly live in aquatic/subaquatic environments, or in decaying matter of the forest [1,5]. They possess water-repellent long hairs with gray or brown coloration that cover their bodies and protect them from most water-borne toxins. Due to low flying abilities, they are often found near their breeding sites. Both larva and adults are important organic matter decomposers. Due to increasing urbanization worldwide, they are commonly found in anthropogenic dwellings such as bathrooms, kitchens, toilets, sewage, poorly maintained drains, or waste dumps [8,9].

### 1.2. Vectorial Role

*Clogmia albipunctata* are not a hematophagous species. Nevertheless, they might pose a health risk associated mainly with the mechanical transmission of various pathogens. *Clogmia albipunctata* may act as a potential mechanical vector of 45 bacterial species mainly associated with nosocomial infections [10,11]. The mechanical transmission can be potentially occurred by contact, secretion, or excretion. Although they do not bite or sting, the remains of their dead bodies may form potential allergens for humans [1]. These pests are a nuisance because they infest in large numbers. Large infestations of drain flies can cause respiratory problems such as allergic rhinitis and asthma due to the possibility of inhaling fine hair-like scales that can fall off their bodies and wings [8]. When they are present in large numbers, they can cause health problems, especially in hospitals. On the other side, drain flies may plug pipes and spread bacteria from the filth they live in, leading to food or water contamination.

### 1.3. Epidemiology and Clinics

**Figure 1:**
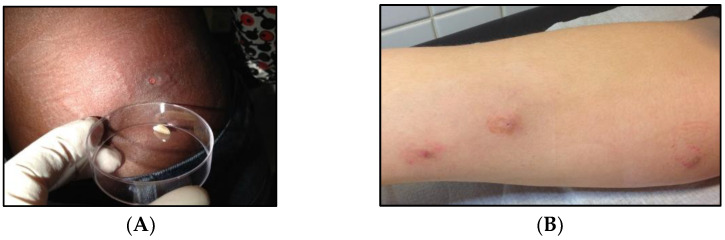
Cutaneous myiasis caused by *Dermatobia hominis* (**A**) and *Cochliomyia hominivorax* (**B**) larvae.

Myiasis is an infection reported to be caused by *C. albipunctata*. It is defined as the infestation of tissues or organ cavities of human and vertebrate animals by Diptera larvae. The lesions develop as a result of larvae feeding on living or dead tissues, bodily fluids, or undigested foods [12]. Myiasis is usually among the five most common dermatologic conditions, representing from 7.3% to 11% of cases [13]. Although it has a worldwide distribution, more etiologic species and greater disease burden occur in tropical and subtropical countries [12]. Poor hygienic conditions and exposed preexisting suppurative lesions are the most predisposing factors for human wound myiasis [10]. Myiasis is commonly classified under three main types: obligatory, facultative, or accidental myiasis [14]. Accidental myiasis is often the result of eating food contaminated with larvae or occurs when a fly lays its eggs in a person’s anus or genital area. Thereafter, the larvae travel toward the rectum or urogenital canal. On the other side, myiasis may be classified according to the body tissue invaded. Cutaneous myiasis is the commonest type of myiasis in humans [15] (see Figure 1). Gastrointestinal and urogenital myiasis, body cavity myiasis, nasopharyngeal, ocular, or aural disorders are other lesser common myiasis [16]. Urethral myiasis is relatively unusual because they are usually protected by clothes which make them often inaccessible to flies. Additionally, most ingested larvae are unable to complete their life cycle in the human digestive system. Dysuria, polycoria, hematuria, nausea, and vomiting are clinical symptoms reported in patients with urogenital and intestinal myiasis [17,18,19,20]. Such complaints are due to inflammatory reactions induced by larvae. Larva toxins cause inflammation and result in pathogenicity [21,22]. The flies belonging to Calliphoridae and Sarcophagidae families are reported as the most myiasis-caused etiologic agents [14,23].

*Clogmia albipunctata* has long been reported as one of the causative agents of accidental myiasis in humans living in poor hygienic conditions with low mobility or ulcerating lesions. It has been frequently recorded in various countries in Asia (Japan [24], India [25], China [26], Palestine [27], Taiwan [28]), Europe (Belgium [2], Croatia [29], Germany [10], Austria [30], Greece [31], Slovenia [32], Slovakia [33], Switzerland [34], and United Kingdom [35]), Africa (Egypt [21] and Morocco [36]), and America (USA [37]).

Regarding the life cycle, bio-ecological criteria, and medical importance of *C. albipunctata* as a non-hematophagous saprophagous insect and considering several articles introducing it as a response to human myiasis, we provided herein a comprehensive review on the epidemiological, entomological and clinical aspects of infections introduced by *C. albipunctata* to understand if this insect is a real etiologic agent of myiasis in human.

## 2. Case Presentation

**Figure 2:**
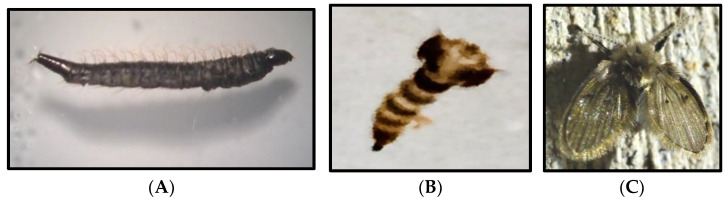
Full grown fourth stage larva (**A**) brought by our patient. Developmental pupa (**B**) and adult (**C**) of *Clogmia albipunctata* obtained by rearing in an incubator at 25 °C and 60% relative humidity.

In February 2017, a 55-year-old French man was referred to the Parasitology–Mycology department of Poitiers hospital (Poitiers, France) for the suspected presence of insect larvae in the mouth. He brought some larval samples of an insect thought to come out of his mouth. Based on the medical records of the patient, no notion of medical or parasitological anomalies was reported. Clinical examination of the patient revealed no lesion or inflammation in the mouth as well as in other body parts. The collected specimens were morphologically identified under a microscope using the identification key of [38] as *Clogmia albipunctata*. They were then reared in an incubator to obtain pupa and adult specimens (Figure 2). Based on the patient’s interrogations, he used the water for teeth brushing collected in a large container used for rainwater collection. He declared that he observed several larvae similar to those he brought for diagnosis in the same container he used for teeth brushing.

## 3. Literature Review

**Figure 3:**
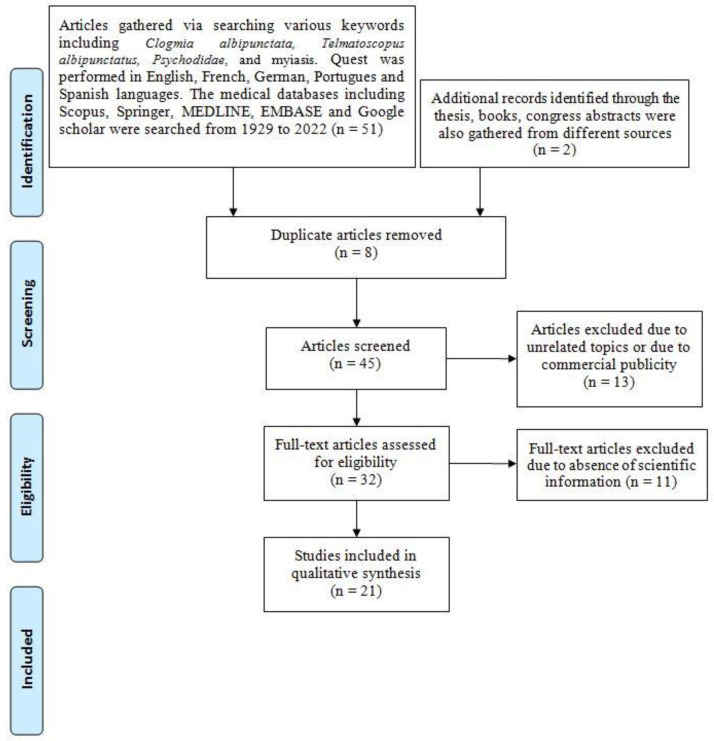
Literature quest strategy used in the present study.

To explore the medical and clinical criteria of myiasis caused by *Clogmia albipunctata*, a literature overview was performed on the released literature, including research articles, case reports, books, and dissertations based on the PRISMA (Preferred Reporting Items for Systematic Reviews and Meta-Analyses) guideline [39]. The articles published from 1929 to March 2022 were explored in various languages using the keywords such as *Clogmia albipunctata*, *Telmatoscopus albipunctatus*, *Psychodidae*, and myiasis in several medical databases, including Scopus, PubMed, Science Direct, ProQuest, Web of Science, Springer, MEDLINE, and Google Scholar (Figure 3). The relevant articles that met the mentioned criteria were selected. Duplicated articles and articles with unrelated topics were excluded. A total of 51 articles published on the mentioned subjects were gathered. Among them, 21 articles that met the study criteria were selected. The detailed epidemiological and clinical features of myiasis cases reported to be caused by *C. albipunctata* are given in Table 1.

## 4. Discussion

Over the past 30 years, *C. albipunctata* has been the subject of a number of developmental studies. This literature review allowed us to evaluate the epidemiological, entomological, and clinical aspects of “myiasis” caused by *C. albipunctata* recorded from 1929 to 2022 and to verify if this insect is really an etiologic agent of myiasis. Among 21 articles reporting *C. albipunctata*-caused human myiasis in the literature, most of them were reported from Asia (11 articles), followed by Africa (5), America (4), and Europe (1) (Table 1). These findings are in accordance with the fact that this insect is more prevalent in tropical and subtropical countries [21,25].

Based on the literature, 12 out of 21 (57.2%) cases were reported as urogenital myiasis, followed by six (28.6%) cases as intestinal myiasis, two (9.5%) as nasopharyngeal myiasis, and one (4.7%) as oral myiasis (Table 1).

### 4.1. Urogenital Myiasis

Urogenital myiasis is relatively more prevalent in patients of tropical or subtropical regions with low socioeconomic status and poor hygienic conditions. It can occur in external (cutaneous) or internal (bladder or urethra) parts of the urogenital track. So far, no species are known to complete a life cycle within the human urinary tract [55]. Poor sanitation and hygiene, limited mobility, chronic debilitating illness, and sexually transmitted infections (STI) are such conditions favoring urogenital myiasis [56].

In an entomological book written by James in 1947 [40], *C. albipunctata* was listed together with other flies to be responsible for accidental urogenital myiasis in humans. Thereafter, other reports of urogenital myiasis were made over 60 years later by Hovius et al. [46], who reported the case of an 18-year-old woman who observed small moving objects in her menses secretions in the bathtub sink after washing herself in the hotel bathroom. No abnormality was observed upon physical examination. Three years later, another case of a patient with the repeated passage of numerous living dark-colored larvae (7–12 larvae) in urine was recorded [21]. Urine analysis and culture were free, and plain X-ray and pelvic and abdominal ultrasound revealed no abnormalities. In 2017, two cases reported by Zhang et al. [26] and El-dib et al. [48] presented the cases of 50- and 24-year-old women who saw the larvae in their urines. The former found three to five larvae in her morning urine, while the latter was a married housewife patient who lived in poor hygienic conditions with the presence of abundant flies in the bathroom. In both cases, the urine tests and urinary ultrasonography revealed no abnormalities. One year later, two additional cases were reported indicating urogenital myiasis in the patients 40 (male) and 28 (pregnant female) years old [25,27]. The latter collected 20 larvae and attributed them to be responsible for urogenital myiasis. However, the relevant investigations were found to be within normal physiological limits. In 2019, three reports of urogenital myiasis were recorded in 24 (unmarried female), 57 (Caucasian male with a history of travels to Iraq and Afghanistan) years old patients as well as in the seven urine samples belonging to the patients with five to twenty-four years old referred to Urology Department [20,49,50]. Except for the first one, all the patients were living in poor hygienic conditions. Unremarkable physical examinations, urinary ultrasonography, and laboratory findings were reported for all of the mentioned cases. Two last cases of urogenital myiasis were reported in Turkey and Iraq in women patients 43 and 34 years old, while the clinical examination and ultrasonography demonstrated no significant findings [52,53]. Concerning Turkish patients [52], the question raised here is how a fly was able to lay eggs in the patient’s urethra, and the larvae could grow to the fourth larval stage without the patient noticing the fly and preventing it from growing. For two married Iraqi patients [53], the detailed interrogation of the patients revealed that the patients dried their underwear in the air. As a result, the flies likely laid their eggs on mentioned tissues, leading to the presence of hatched eggs and larvae on the underwear of the patients. Nevertheless, it does not imply myiasis in mentioned patients.

Of 12 cases of urogenital myiasis, most of them were women whose larvae were observed only in their urine based on the patients’ declaration, and none of them were isolated by the attending physician or by the patient from the urogenital lesion (Table 1). In most of the cases, the physical examination and ultrasonography demonstrated no significant abnormalities. Moreover, except for larva/adult fly’s photo in some cases, no additional evidence such as the lesion photo, imaging, or clinical or parasitological evidence were provided by the authors to confirm firstly the presence of the lesion (myiasis), and in further step, isolating *C. albipunctata* larvae from mentioned lesion as a causative agent [20,21,25,26,27,40,46,48,49,50,52,53]. In none of the mentioned reports, any information about the urine sampling conditions (e.g., direct or indirect sampling from the urinary tract, sampling site, sterile or contaminated containers) was given. Furthermore, no photo or imaging demonstrating direct isolation of *C. albipunctata* larvae from external or internal urinary lesions was provided. Due to the fact that the urinary tract is usually covered, how can a fly lay eggs in the urinary tract without the patient noticing? Other members of Psychodidae family with similar life cycle such as *Psychoda albipennis* were also reported as responsible for urogenital myiasis in humans [55]. Regarding the similar living environment and non-hematophagous and non-carnivorous nature of these flies, no supportive evidence implying to human myiasis was given in the mentioned reports. As an unusual or atypical case, the myiasis caused by larvae of flower flies such as *Palpada sovtellaris* was reported in the literature as well [57]. However, the questions raised in these reports about the infection way, breeding, and immature developmental stages of causative insects, without the patient noticing, always remain unanswered. Among the patients who presented the symptoms such as dysuria, periurethral itching, pollakiuria, or abdominal pain with micturition sensation, no evidence of lesion was given. The presence of larvae in the urine stated by the patient, together with the lack of supportive clinical signs or lesions in the urogenital tract, cannot affirm myiasis caused by *C. albipunctata*. Furthermore, there is no way to track the source of the infestation in order to determine if the larvae were isolated from the urogenital tract of the patient or simply collected from the patient’s toilet bowl.

### 4.2. Intestinal Myiasis

Intestinal myiasis is a condition when the fly larvae inhabit the gastrointestinal tract and are passed out in feces via contaminated food or water. It can be found throughout the world but is more common in regions with low socioeconomic conditions and poor hygiene [58]. Children and elders are more susceptible to intestinal infection.

One of the first cases of intestinal myiasis was reported by Tokunanga et al. [41] in a 48-year-old woman who ingested the *C. albipunctata* eggs and was thought to be developed in the intestine. Nevertheless, the authors believed that it was highly improbable that fully grown larvae or pupae could be ingested without being seen by the patient. Zumpt [42] rejected the hypothesis that the ingested larvae could live as facultative parasites in the alimentary tract and believed that the larvae are continually ingested with polluted food. Smith and Thomas [45], in a short text, reported the case of a seven-year-old male child vomiting a number of fly’s larvae with no supplementary clinical and parasitological details. Two decades later, Tu et al. [28] reported the case of human intestinal myiasis caused by larvae of *Telmatoscopus albipunctatus* in a 21-year-old male patient who experienced gastroenteritis and anal itching. Five living larvae were passed out in the patient’s feces. It was supposed that the patient was likely infected by ingestion of eggs or early-stage larvae in contaminated food. Mokhtar et al. [47] reported the case of a 41-year-old female Malaysian patient who found the “worm” in the feces after consuming a meal in an area infested by flies. Five years later, the case of a 36-year-old married male was documented by El-Dib et al. [51] in Egypt with abdominal pain, diarrhea, perianal itching, irritability, and insomnia for one year. The endoscopy revealed no abnormal findings. The authors identified the fourth stage larvae in the stool brought by the patient as *C. albipunctata*. However, the authors did not provide any explanation as to what condition the patient’s stool was prepared for and whether it was probably due to the toilet that the patient uses.

Of six cases of supposed intestinal myiasis [28,41,42,45,47,51], two complained of abdominal pain and diarrhea, two reported no clinical symptoms, and one little boy was reported to have vomited *C. albipunctata* larvae. Nevertheless, the authors did not provide credible evidence of an obvious lesion in the digestive system supported by imaging or parasitological examinations. The authors were satisfied with the stools brought back by the patients without giving additional information about the origin and conditions in which the stools were collected. Similar to urogenital cases, most intestinal myiasis reports suffer seriously from the lack of reliable clinical or parasitological supportive evidence implying lesions or myiasis in the patients. Furthermore, the authors did not explain the source and mode of infestation in the reported patients.

### 4.3. Nasopharyngeal Myiasis

Nasal myiasis is the infestation of the nasal cavity by dipterous larvae and which are commonly documented in developing countries where sanitation is a public problem [59]. Atrophic rhinitis, diabetes, and nose-involved malignancy are the susceptible conditions where nasopharyngeal myiasis can occur.

The first case of nasopharyngeal myiasis was reported by Nevill et al. [43], presenting an elderly Pretoria woman who brought two psychodid third-instar larvas discharged from her nose into the water. Another nasopharyngeal case was recorded seven years later by Mohammed and Smith [44] in a short editorial letter. They reported the case of a European employee of Ahmadu Bello University who brought a larva that claimed to come out of his nostrils. He used the collective water of an open tank for washing his face. Surprisingly, the authors stated that the larva’s intestine contained dark-bluish blood, indicating the larvae had been feeding on the mentioned patient. In both cases, the diagnosis was performed based on the statement of the patient discharging likely the larvae from the nose. Both reports suffer from the lack of supportive imaging or photo demonstrating the lesion as well as the isolation of fly’s larvae from nasopharyngeal myiasis. Furthermore, no information on the source and mode of infestation, patients’ reaction to egg laying of *C. albipunctata,* or consequence clinical reactions (e.g., inflammatory lesion) related to the presence of larvae in the nose was provided.

### 4.4. Oral Myiasis

Oral myiasis is a rare pathology in humans associated with poor oral hygiene, trauma, alcoholism, and suppurating lesions [60].

Based on the literature, a single case of oral myiasis has been reported in which the authors [54] stated the appearance of four *C. albipunctata* mature stage larvae in the residual dental root of a 26-year-old woman after injecting a normal saline solution. Two active living larvae were also found during teeth brushing by the patient. The presence of final stage larvae implies entrance and laying eggs by the female flies, hatching, emergence of newborn larvae, and their development to further stages in the patient’s mouth or points to consumption of contaminated water. If the patient’s statements are correct, the question that arises is how the patient did not notice the fly entering and laying eggs at her teeth root. Given that the patient was brushing once a day, how were the eggs still present in the patient’s mouth? Whether the inflammation could really be attributed to myiasis or whether it was related to the residual root itself, which could lead to chronic inflammation. Additionally, how can larvae stick to the roots of teeth and survive? How were the eggs and larvae not swallowed and digested during eating or drinking by the patient? The authors did not give any detail about the infestation way or the source of water used by the patient for teeth brushing. No imaging or photos certifying the presence of larvae in the patient’s mouth was provided. Regarding the occurrence of oral myiasis commonly among patients with poor hygienic conditions or open-mouth sleeping habits, no notion of mentioned conditions was reported by the authors.

In the oral infestation we presented in this article, the patient’s interrogations revealed the contaminated water used for teeth brushing as a source of infestation. Furthermore, no notion of lesion or myiasis was recorded in our patient.

According to bio-ecological criteria, most of the patients discovered the moth flies in their toilet or bathroom. This is in agreement with the most frequently reported habitats of *C. albipunctata* [50]. Regarding the fact that the larvae commonly feed on the decaying organic matter with a siphon for breathing in the aquatic areas and the adults feed on nectar or other liquid carbohydrates, they do not feed on human or animal tissues in any stage of their life cycles. Furthermore, *C. albipunctata* has never been described as a carnivorous or hematophagous insect.

In Table 1, the “larvae isolation/observation” was given for the aforementioned 21 reports. Except for one report [54], in none of them the flies’ larvae were isolated from the lesion by the patient or physician. Moreover, there is any imaging or photo certifying the myiasis lesion and direct isolation of the larvae. In most cases, these parasito-clinical examinations were missed or misinterpreted in the articles introducing this insect as an etiologic agent and mostly concentrated on the fly’s larvae identification instead of demonstrating the myiasis lesion and the larvae within these lesions. Surprisingly, there is even no notion of “wound” or “lesion” in most of them, which is the least characteristic of the definition of myiasis. Concerning mentioned single case [54], any larvae within the lesion were demonstrated in the photo of this report as well.

Delusional parasitosis is a psychological disease or phobia in which individuals have a persistent belief that they are infested with living or nonliving pathogens such as parasites, insects, or bugs, while no such infestation is really present [61]. Therefore, it is not entirely out of the question for some patients who are diagnosed with *Clogmia albipunctata*-caused myiasis to suffer from mentioned disease and to present to the treating physician the insects they have found in the bathroom or toilet as the causal agent of the disease.

Due to the poor flight ability of drain flies, locating their immature developmental sites is often easy because the adults are commonly found near their breeding sites. However, the flies can develop in any area with standing water and organic material. Therefore, the simplest way to control drain flies is by manually removing the organic material in the drain where eggs are laid and larvae feed. Because of their attraction to light, drain flies can be tracked by using fan bait-based traps in visible or ultraviolet light. However, killing only adult flies is usually ineffective; larval food sources must be simultaneously removed to inhibit egg laying by females [12].

## 5. Conclusions

*Clogmia albipunctata* was reported for a long time as an etiologic agent of myiasis in humans. The absence of a precise definition of myiasis and the lack of incontestable epidemiological, entomological, and clinical evidence in the articles introducing *C. albipunctata* as a causative agent led us to doubt its role in myiasis in humans. Herein, we highlight the importance of indisputable parasito-clinical examinations demonstrating the lesion caused by *C. albipunctata* larvae (myiasis) and documenting these parasito-clinical examinations by supportive imaging. Therefore, it is necessary to take into account this misinterpretation and make an accurate diagnosis based on the isolation of insect larvae from the corresponding lesion instead of a diagnosis only according to the flies’ larvae identification and dubious statement or observation.

## Figures and Tables

**Table 1:** Epidemiological criteria and clinical features of the myiasis reported to be caused by Clogmia albipunctata in the literature.

Author(s)	Epidemiological Criteria			Clinical Features				References
	Country	Age	Sex	Clinical Manifestation	Location	Larvae Isolation/Observation *	Treatment	
James 1947	USA	NA	NA	NA	Urogenital	NA	NA	[40]
Tokunanga et al. 1953	Japan	48	F	NA	Intestinal	NA	NA	[41]
Zumpt 1965	Germany	NA	NA	NA	Intestinal	Ingested food	NA	[42]
Nevill et al. 1969	Pretoria	Elderly	F	Movement felt in nose	Nasopharyngeal	Observed in discharged nose excretion	Application of insecticide strips for one month	[43]
Mohammed and Smith 1976	Nigeria	NA	M	Irritation in nose	Nasopharyngeal	Broughted by the patient	NA	[44]
Smith and Thomas 1979	Malaysia	7	M	Severe gastro-enteritis	Intestinal	Observed in the vomiting	NA	[45]
Tu et al. 2007	Taîwan	21	M	Abdominal pain and anal itching	Intestinal	Observed in stool sample	Anthelmintic mebendazol	[28]
Hovius et al. 2011	Netherland (Mexico)	18	F	No symptom	Urogenital	Observed in menses secretions	NA	[46]
El-badry et al. 2014	Egypt	NA	F	Dysuria, fever and itching in the periurethral and genital regions	Urogenital	Observed in urine sample	Antibiotic and antiseptic treatment	[21]
Mokhtar et al. 2016	Malaysia	41	F	No symptom	Intestinal	Observed in stool sample	Albendazole	[47]
El-dib et al. 2017	Egypt	24	F	Dysuria, pollakiuria	Urogenital	Observed in urine sample	Ivermectin	[48]
Zhang et al. 2017	China	50	F	Frequent micturition and urgency	Urogenital	Observed in urine sample	Broad-spectrum antibiotics for one week	[26]
Hjaija et al. 2018	Palestine	28	F	Abdominal pain with burning sensation in urinating	Urogenital	Observed in urine sample	Antibiotic and antiseptic therapy, pleniful hydratation	[27]
Sarkar et al. 2018	India	40	M	Lower abdominal pain with painful burning sensation during micturition	Urogenital	Observed in urine sample	Fluroquinolone	[25]
Geremy-Depatureaux et al. 2019	Canada	57	M	No symptom	Urogenital	Observed in urine sample	Ivermectin	[49]
Fatima et al. 2019	India	24	F	No symptom	Urogenital	Observed in urine sample	Ivermectin	[50]
Farrag et al. 2019	Egypt	5–24	M&F	Dysuria, frequency of micturition and genital pruritus	Urogenital	Observed in urine sample	Antibiotics and Ivermectin	[20]
El-dib et al. 2020	Egypt	36	M	Abdominal pain, bloating sensation, diarrhea intermittent with constipation, perianal itching, irritability and insomnia	Intestinal	Observed in stool sample	Ivermectin and Nanozoxide twice daily	[51]
Gökçe et al. 2020	Turkey	43	F	No symptom	Urogenital and gastrointestinal	Observed in stool and urine samples	NA	[52]
Alshimmre and Ismail 2020	Iraq	34	F	Mild dysuria	Urogenital	Laying eggs by female flies on the underwears	Antibiotics	[53]
Chen et al. 2022	China	26	F	No symptom	Oral myiasis	Isolated from residual dental root	Antibiotics and Ivermectin	[54]

F: Female; M: Male; NA: Not Available; *: Observed by patient or isolated by treating physician.

## Data Availability

Not applicable.
